# Medicine in the early twenty-first century: paradigm and anticipation - EPMA position paper 2016

**DOI:** 10.1186/s13167-016-0072-4

**Published:** 2016-10-25

**Authors:** Olga Golubnitschaja, Babak Baban, Giovanni Boniolo, Wei Wang, Rostyslav Bubnov, Marko Kapalla, Kurt Krapfenbauer, Mahmood S. Mozaffari, Vincenzo Costigliola

**Affiliations:** 1 European Association for Predictive, Preventive and Personalised Medicine, Brussels, Belgium; 2 Radiologic Department, Rheinische Friedrich-Wilhelms-University of Bonn, Sigmund-Freud-Str. 25, 53105 Bonn, Germany; 3 Breast Cancer Research Centre, Rheinische Friedrich-Wilhelms-University of Bonn, Bonn, Germany; 4 Augusta University, Augusta, GA USA; 5 Department of Surgery, School of Medicine, Augusta University, Augusta, GA USA; 6 Dipartimento di Scienze Biomediche e Chirurgico Specialistiche, Università di Ferrara, Via Fossato di Mortara, 64A, 44121 Ferrara, Italy; 7 Institute for Advanced Study, Technische Universität München, Garching bei München, Germany; 8 School of Medical Sciences, Edith Cowan University, Perth, Australia; 9 Beijing Municipal Key Laboratory of Clinical Epidemiology, Capital Medical University, Beijing, China; 10 WHO Expert Panel (Member), Geneva, Switzerland; 11 Global Health Epidemiology Reference Group (GHERG), Edinburgh, UK; 12 Clinical hospital “Pheophania” of State Affairs Department, Kyiv, Ukraine; 13 Zabolotny Institute of Microbiology and Virology, National Academy of Sciences of Ukraine, Kyiv, Ukraine; 14 European Medical Association, Brussels, Belgium

**Keywords:** Traditional complementary alternative medicine, Person-centred medicine, Individualised medicine, Stratified medicine, Personalised medicine, Precision medicine, Predictive, preventive, and personalised medicine, Advantage, Limitation, Implementation

## Background

Challenges of “standardisation” and “individualisation” have always been characteristic for medical services. In terms of individualisation, the best possible individual care is the ethical imperative of medicine, and it is a good right of any patient to receive it. However, in terms of standardisation, all the available treatments are based on guideline recommendations derived from large multicentre trials with many thousands of patients involved. In the most optimal way, the standardisation and individualisation should go hand-in-hand, in order to identify *the right patient* treating him/her with *the right medication* and *the right dose* at *the right time point*!

Further, in paradigm and anticipation, there is a big discrepancy between “disease care” and “health care” which dramatically impacts ethical and economical aspects of medical services.

Several approaches have been suggested in ancient and modern medicine to conduct medical services in a possibly optimal way. What is the difference amongst all of them and how big is the potential beyond corresponding approach to satisfy the needs of the individual, the patient, professional groups involved and society at large?

On behalf of the “European Association for Predictive, Preventive and Personalised Medicine,” the dedicated EPMA working group provides a deep analysis in the issue followed by the expert recommendations considering the multifaceted aspects of both “disease care” and “health care” practices including ethics and economy, life quality of individuals and patients, interests of professional groups involved, benefits of subpopulations, health care system(s) and society as a whole.

## Traditional, complementary and alternative medicine (TCAM)

TCAM, also called “integrative medicine,” is considered as an amorphous concept comprising a range of ancient, long-standing but still evolving treatment approaches being practised mainly in their countries of origin as well as in countries into which corresponding expertise has been “imported” [1]. TCAM refers to health practices, approaches, knowledge and beliefs incorporating plant-, animal- and mineral-based medicines, spiritual therapies, manual techniques and exercise (e.g. in form of acupuncture, dietary therapy, herbal medicine, moxibuston, TaiJi, Ayurveda, amongst others) applied singularly or in combination to diagnose, treat and prevent illnesses or maintain well-being [2]. However, the educational level of the doctor is critical for the quality of TCAM that depends on the national/local curricula varying substantially from country to country and, therefore, may not be adequate enough to fully realise potential benefits of various forms of TCAM modalities. TCAM approaches are frequently considered as being non-evidence based [3]. Further deficiencies arise from evident philosophical and religious differences as well as some cultural barriers between the countries of origin and countries into which TCAM is “imported” [1]. Nevertheless, in addition to the conventional medicine, TCAM is getting more and more popular and well-pursued in Western countries. From view point of predictive and preventive medicine, TCAM provides a unique expertise for recognising the so-called suboptimal health conditions before a clinical manifestation of severe pathologies [3–5]. These global trends make particularly attractive consideration regarding innovative hybrid approaches which would utilise advantages of both TCAM and modern medicine and, therefore, benefiting patients and enriching the spectrum of tools and overall expertise of the dedicated professional groups assuring the reproducibility of TCAM technologies and outcomes [6]. However, those approaches are currently underdeveloped and require additional major efforts in terms of multi-professional collaboration, scientific and technological discoveries and extensive financial support.

## Person-centred medicine

The main idea of the person-centred medicine is to promote health and, therefore, reduce disease burden. In this concept, any health condition is considered as an individual state of physical, mental, social and spiritual well-being. Contextually, health care approaches are prioritised by person-centred medicine compared to a disease care. Humanistic interpretation of medicine is characteristic including the articulation of science, enhanced understanding of positive health versus illness, emphasised personalisation of all medical services as well as strong patient empowerment and essential responsibility of every person, at individual and community levels. “All for one and one for all”—a smart but perhaps a bit naive slogan introduced by the Three Musketeers fits well to the philosophy of the person-centred medicine. Therefore, a realisation of those ideas demands clear definitions and validated strategies to reach a reasonable level of maturity in health care [7].

## Individualised medicine

A great strength of individualised medicine (IM) is to provide a holistic and integrative approach for medical care. IM comprises curative, rehabilitative and preventive examination as well as treatment methods customised for the individual and the patient [8]. IM well recognises a multidimensional interaction of internal and external risk factors, genetic background, age, gender, environmental risk factors, lifestyle, culture and beliefs as well as social status in the overall predisposition of individuals to specific diseases, the disease development, the natural course of disease and the response to therapeutic intervention. These factors vary from individual to individual. Contextually, IM aims to categorise patients into clinically relevant subgroups (that is the content of the “Stratified medicine” —see below). Hence, at the heart of the concept of IM is a stratification that “individualises” a one-size-fits-all standardised intervention into a group-specific intervention. Less clear concepts and approaches are provided by IM towards “predictive and preventive medicine”—see below.

## Stratified medicine

Stratified medicine means looking at large groups of affected individuals (e.g. cancer patients) to try and find ways of predicting which treatments/patient sub-types are likely to respond to. Specifically in cancer, it involves looking in detail at the cancer cells and their genetic make-up. The purpose of the approach is to find out which treatment algorithms are more likely to work [9]. Patient stratification is one step towards individualised patient treatments and so-called “personalised medicine”—see below.

## Personalised medicine

The term “personalised medicine” is the keyword to refer to the best possible, most optimal and innovative medical approaches in the early twenty-first century, to justify grant applications and to receive dedicated budgets. However, in order to make anticipation by personalised medicine as realistic as possible, this term should be pragmatically sub-divided into its clear subcategories, namely “semi-personalised” versus “true personalised” as it has been discussed and published elsewhere in scientific literature [10].

“Semi-personalised medicine” compromises between standardisation and individualisation in medicine, the first step of which is the stratification of big patient-groups according to certain well-known characteristics (e.g. specific biological characteristics of the tumour). In the next step, individual patients within the group are treated according to the algorithms adapted to the entire stratified group. Consequently, the treatment efficacy varies from patient to patient within the group, since a limited number of characteristics in common is considered by the treatment algorithm; all other individual characteristics are not taken into account but may sufficiently impact individual outcomes.

“True personalised medicine” is based on the “individual patient profile” (see “Predictive, preventive and personalised medicine (PPPM)” section) directing to a tailored therapy that maximises the efficacy for that one patient in particular.

However, a disadvantage of “personalised medicine” is that its contents are adapted to the needs of disease care for treatments of diseased individuals and individual patient cohorts but not for health care of individuals to maintain in a good mental and physical shape avoiding clinical manifestation of diseases.

## Precision medicine

The terms precision, personalised and individualised medicines are often used interchangeably [11]. Precision medicine is a concept of therapeutic and preventive modality for disease that takes into account individual variability in genes, environment and lifestyle. It refers to the tailoring of medical treatment to the individual characteristics of each patient [12]. Precision medicine is considered a relatively new approach in disease and health care, although it has been around for a while and has limited application in certain fields of medicine such as blood transfusion and organ transplantation. Further, precision medicine faces a number of serious challenges that need to be addressed [13–18] as summarised below.

- Knowledge gap: Extensive and costly long-term education is required for all health care system authorities, physicians and participants to fully understand the dynamic and potential objectives of precision medicine.
- Authority and interpretation: Even for the field specialists, an interpretation of DNA data for individual health outcomes remains sophisticated and the problem of interpretability continues to grow. Consequently, many doctors are simply not able to make sense of genetic tests and to communicate the results accurately to their patients.
- Data storage: Gene sequencing of an individual produces *massive* amounts of data. The sequencing of thousands, if not millions, of people will produce unimaginable amount of data. How will we store the data and effectively analyse to derive useful information and to interpret the data?
- Pathogenic mechanism: Many diseases have complex and multifactorial pathogenic mechanisms which would make it very difficult to identify a specific gene responsible for their manifestations.
- Most technologies and equipment required for effective implementation of precision medicine are still in embryonic stages.
- Privacy/security: Cyberattack is increasingly a major hurdle to maintaining privacy and security for all particularly given the current state of world affairs. Such valid concerns are already well-recognised for economic, energy and defence sectors across the globe. Precision medicine relies on massive public and personal data requiring sophisticated and extensive infrastructure and technology. Thus, vulnerability due to breach of security and privacy violation could have devastating consequences for the successful implementation of precision medicine [19] and data misusing, e.g. for economic and political purposes with a consequent discrimination of affected individuals and even (sub)populations involved in the database containing sensitive genetic information and family history amongst others.
- Coordination and policies: For precision medicine to have its greatest impact, federal and private health insurance companies have no option but to become comfortable with value-based drug pricing.
- Variability of phenotypic features in population: It is difficult, if not impossible, to detect, decipher and utilise phenotypic characteristics of every individual as indicators for diseases as seems to be proposed by precision medicine.
- Data relevance: The usefulness of data gathered from smaller groups may not be sufficient to make larger population health recommendations.
- Culture: Prevention of abuse of information for unintended purposes such as screening potential partners and denying insurance coverage is a serious concern. How will this affect the culture? Will we be cultivating a different kind of racism, on a genetic basis?
- Ownership: Who will have ownership of the data? Will it be the government? It is noteworthy that the FDA has blocked companies from allowing individuals to have access to their own genetic information. Will this change as part of the new initiative?
- Compliance: There is no binding protocol to guarantee that all individuals would follow the recommendations made by precision medicine so the diseases could be prevented, controlled or cured.
- Drug/device industry: Genetic research and development of treatment options have been very promising and productive in the private sector. How will government involvement affect research? Will governmental agencies work cooperatively with them or competitively?
- Diversion from overarching goals of health care system: The focus and concentration of human and financial resources on precision medicine may divert attention and concerns of health care system from efforts to remedy the foundational causes of ill health such as poverty, obesity and education.
- Health care costs: Genetic mapping of a population, and analyses of data, securing the information and deriving treatment recommendation are very costly which can be readily hampered by budgetary constraints of high dynamic economies. Further, the costs of converting the intellectual capital to therapeutic modalities must also be taken into account and built into the health care system.
- Finally, it is noteworthy that the concept of precision medicine may be a repackaging of the ideals advanced by the human genome project in 2000. The hope was to identify genetic markers for the ultimate objective of developing novel biomarkers and overcome perceived therapeutic deficiencies and overcome the pressing issue of non-responders. However, this noble objective has not been fully materialised yet, leading one to the question: Is precision medicine “old vine in a new bottle”?

**Fig. 1:**
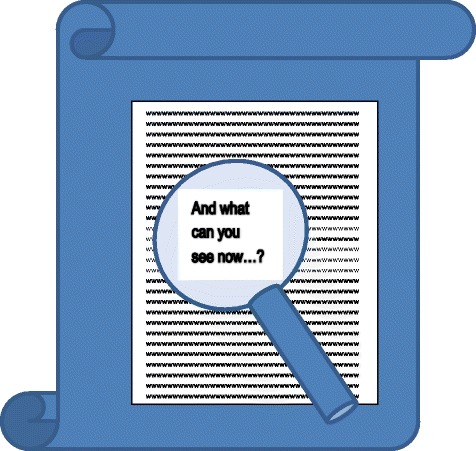
“Precision” itself does not guarantee for better understanding of an issue. An old wise dictum adopted by all European cultures/languages warns—*one cannot see the forest for the trees*. Hence, technologically driven higher resolution of individual elements does not automatically mean that you can better recognise the complex problem which you are looking for, particularly when the zoomed element (the tree) is prioritised and/or pulled out from the overall context or zooming itself makes the complete picture (the forest/multifactorial issue) unreadable. Contextually, better understanding of the complexity in medicine is not guaranteed by “precision medicine” itself. Advanced health care demands a close cooperation between all issue-related fields, integration of multidisciplinary knowledge and innovative technologies based on long-term strategies and concepts considering interests of patients, professionals and society at large

In conclusion, precision medicine could potentially improve preventive methods and therapeutic options. However, a number of challenges remain as alluded to above—see also the cartoon in Fig. 1. Precision medicine will have to demonstrate consistency, coherence, comprehensiveness, clarity and relevance to every individual and community impacted by these developments in medical research and treatment. Will precision medicine deliver all that it promises? With increasing shrinking of financial resources, is it wise to invest millions of dollars/euros in an approach for which its risks versus benefits ratio does not ring a satisfactory bell? Perhaps, stakeholders and authorities would be better off to further invest and focus on the already established concepts of “predictive, preventive and personalised medicine.”

## Predictive, preventive and personalised medicine (PPPM)

### The paradigm shift from “unPPPM” to “PPPM”

The above described great plurality of approaches indicates a broad understanding of clear deficits which do exist in currently applied medical services and attempts of diverse professional groups to remedy the deficits. On the other hand, there is an increasing level of understanding that persisting deficits carry a fundamental character and, therefore, cannot be solved by superficial modifications of health care systems facilitating individual technologies such as “cancer genomics” by “precision medicine.”

Global deficits are well-defined and described elsewhere as *unpredictable, unpreventable and impersonal medicine* [20, 21]. It is evident that a paradigm shift is needed to move from “reactive” to “predictive, preventive and personalised medicine” as a new philosophy covering both “health care” and “disease care”, promoting an integrated approach combining advantages of individual bio/medical fields and technologies and consolidating a multi-professional collaboration.

**Fig. 2:**
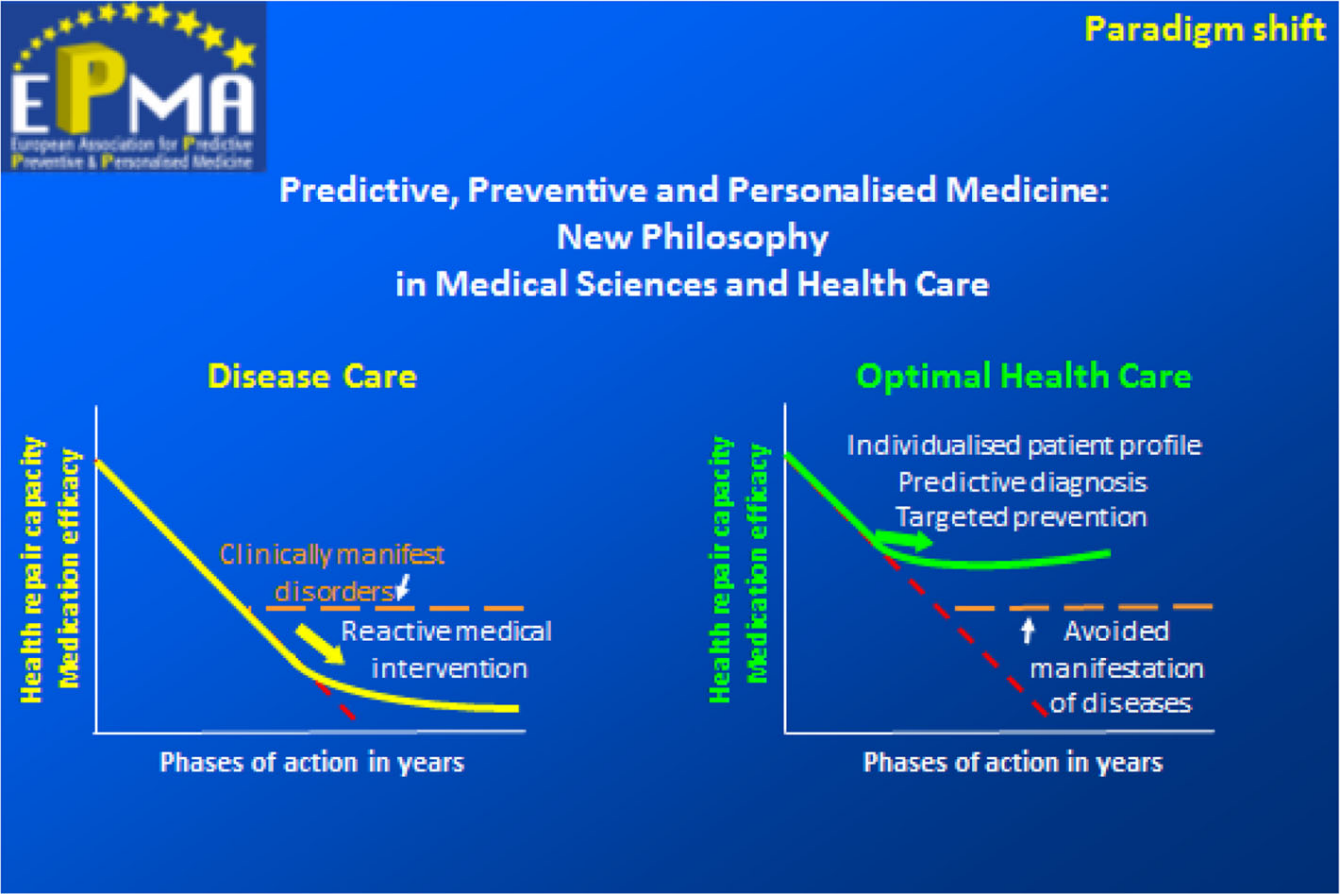
Paradigm shift from “reactive” to “predictive, preventive and personalised medicine”

New paradigm has been created by the EPMA experts as published earlier [22]—see Fig. 2.

### Particular emphasis on ethics in PPPM

Sometimes, we, living in the XXI century, forget what was taught when the first universities were established at the birth of XI century: “Never discuss about names,” “Never enter an onomatomachia”, that is, never enter a fight (in ancient Greek, *μάχη*, *máchi*) about names (in ancient Greek, *όνoμα*, *ónoma*). Sometimes, the meanings of the names “person-centred medicine,” “individualised medicine,” “stratified medicine,” “precision medicine,” “personalised medicine,” etc. are not so sharp; sometimes, they intersect, but it is not clear until which point.

This is not a void and abstract “philosophical” question (actually, over the centuries and the millennia, the philosophers never thought that a question of names was a genuine philosophical question). This is a matter of money, a matter of grants, and a matter of power. This means that the onomatomachia now occurring in the field of contemporary biomedicine is actually a war for money and for power. But the citizens, in particular a subset of them, that is, the diseased citizens, are totally disinterested about it, even if, unfortunately, negative side effects of this war affect them. Citizens are interested in a *personalised care*, whatever this could precisely mean [23].

This means that beside a scientifically well-founded medical approach facing their *unique* potential or actual disease, they wish that their *unique* biography could be taken into account as well. Yet let us put aside for a while the biographical part (i.e. the age, gender, cultural, ethnic, religious, socio-economic diversity) of an actual or potential patient, even if we know, from epigenomics, that patients’ lifestyles and the environments in which they live are extremely impacting their quality of life and their actual or potential diseases. Let us focus on the “medical” part.

A citizen with an actual or potential disease wants a medicine in which he/she is at the centre, a medicine which is tailored on his/her polymorphism, a medicine which is able to provide him/her with the right therapy, in the right dose, at the right moment, for the right period of time. But he/she also wants a medicine which is able to predict and prevent possible diseases. He/she is not interested in the way in which this kind of medicine is called. But he/she is interested in understanding why it is called in that way, in order to appreciate its potential ability to restore health. And this is the real advantage of speaking in terms of *predictive*, *preventive and personalised medicine*: the actual or potential patient understands what is going on!

Nevertheless, there is something more. The PPPM lends itself to an over-arching umbrella under which the main ethical issues of contemporary biomedicine could be positively tackled. Certainly, a predictive and preventive approach could imply several ethical problems linked, for example, to overdiagnosis and overtreatment, detection of incidental findings, psychological burden and severe existential choices connected with the knowledge of the probability of a possible disease affecting us or our offspring and lineages, or connected with our reproductive choices, etc. Contextually, PPPM plays a crucial role as the optimal medical partner of a serious ethical counselling (and here, the patient’s biography plays its main role) offered to actual or potential individual patients, in order to empower them to make an infirmed choice about the diagnostic, surveillance or therapeutic path to take, especially whenever these paths intersect ethical or existential problematic situations that they have to solve [24].

### Towards scientific excellence and practical PPPM implementation: special professional focuses by the EPMA

This subsection is based on the well-elaborated PPPM aspects evidently advancing medical sciences and health care. Corresponding professional statements have been approved by the association within the fundamental document resulting from the EPMA Summit 2014 under the auspices of the presidency of Italy in the EU [6].

#### PPPM in cancer: the key questions puzzling medical sciences and advancing health care

The majority of people may carry hardly detectable micro- and asymptomatic tumour lesions as it has been demonstrated by a series of detailed autopsy studies. However, those lesions do not necessarily progress into clinically manifest oncologic diseases. Furthermore, there is a phenomenon of the so-called metastatic inefficiency, due to less than 1 % of all disseminated and circulated tumour cells which have a potential to form secondary and distanced tumours (metastatic disease) [25]. Contextually, the key question puzzling modern predictive, preventive and personalised medicine in oncology is how to predict and effectively protect against clinical manifestation of the disease by distinguishing between “silent” carriers of tumour lesions and patients who are predisposed to a disease development and progression. The clue might be a “fertile” microenvironment that effectively supports the tumorigenesis, tumour invasiveness and aggressive metastatic disease [26]. The mechanisms “fertilising” the microenvironment for the cancer advancement are well-addressed by innovative PPPM strategies in cancer [26–29].

#### PPPM advancements in CVD management: a global health issue

Currently, the CVD-related health burden is the most severe in developed countries and becomes overgrown in developing countries as well. The main reason for that is that the chronic disease stages, multifactorial diseases and comorbidities are not adequately addressed, since they do not follow the PPPM principles in currently practised health care systems [30]. An advanced CVD management is needed at both population and individual levels considering complex cardiovascular risk factors, co-morbidities, individualised patient profiles, optimised screening programmes and innovative preventive strategies. Chronic suboptimal health conditions such as primary vascular dysregulation (Flammer syndrome) may be relevant for a number of predispositions and severe pathologies with poor outcomes [31–33]. Consequently, the promotion of PPPM in CVD management is a global health issue [34–36].

#### Global epidemic of diabetes type 2—twenty-first century disaster and PPPM solutions

Current epidemiologic studies report about over 400 million of diabetes mellitus (DM) diseased patients worldwide. A big portion of DM cases remains undiagnosed. More and more teenagers are affected by DM type 2. The global epidemic of DM type 2 places an alarming burden on health care systems. The consequent challenges and costs overload both developed and developing countries and economies. An effective implementation of PPPM concepts to diabetes care is due long ago. EPMA emphasises the need to address the all-encompassing complex approach for population screening, primary, secondary and tertiary care benefiting non-diseased individuals, predisposed subgroups and affected patient cohorts including those with comorbid pathologies such as CVD, cancer, and neurological, neuropsychiatric and neurodegenerative diseases (NNND) amongst others [6, 35, 37, 38].

#### PPPM advancing the comprehensive area of NNND

In a very few years, NNND are predicted to represent the majority of socially and economically devastating disorders and diseases. Multifactorial physical and cognitive disability of NNND-affected patient cohorts results from individual interplay of genetic, epigenetic and environmental risk factors. Contextually, the comprehensive area of NNND demands new strategies which would create a robust platform for the cost-effective medicine of future NNND management [39–42]. Consequently, the advanced PPPM concepts do place particular emphasis on primary prevention by the identification of predisposed individuals, improved patient stratification and treatments tailored to the person [6]. However, new regulations and innovative reimbursement programmes are mandatory to prompt an effective implementation of the above listed concepts.

#### Rare disease (RD) management: *proof-of-principles* for personalised medical care

Although an entire spectrum of RDs affects many millions of people worldwide (e.g. in Europe, there are at least 30 million patients), currently, no appropriate diagnostic and treatment approaches are available for most of afflicted with individual RDs. The majority of RDs can be diagnosed in prenatal and early postnatal periods. Due to the genetic background of most RD pathologies, the multimodal diagnostic and treatment approaches propagated by PPPM are instrumental for personalisation of RD management [43].

#### Ancient medical traditions “reinforced” by innovative PPPM concepts

PPPM creates a unique platform for “reinforcing” traditional approaches of the ancient medicines (TCAM). PPPM-TCAM hybrid demonstrates a great potential in person-centred and participatory medicine, disease prediction in individuals with suboptimal health condition, targeted prevention and individualised treatments. If properly designed, PPPM-TCAM approach may be of particular value for health care systems that empowers communities and individuals [3, 44].

#### Application of PPPM to the pain management benefiting all medical fields

Pain management is the central issue for a variety of syndromes, acute, chronic and systemic disorders. Pain diagnostics and treatment are highly individual involved in a wide spectrum of suboptimal health conditions, early and advanced stages of developing pathologies and collateral diseases such as CVD, NNND, diabetes, and cancer. Application of PPPM concepts to advanced pain management demands multidisciplinary expertise considered in the context of improved health care economy and policy and direct benefits to the patient [45–47].

#### Impacts of the oral and dental health: novelty by PPPM concepts

On the one hand, dental diseases are frequently caused by systemic disorders such as diabetes mellitus. On the other hand, dental and oral pathologies are both early indicators and risk factors for a variety of multifactorial diseases. This includes pre-term birth, a spectrum of vascular pathologies, stroke, heart and lung disease, diabetes mellitus with comorbidities, some types of cancer, neurological disorders and several mental disorders such as depression, anxiety, anorexia and even bulimia. Therefore, investigation of the cause-and-effect relationships between oral and dental diseases on the one hand and multifactorial systemic disorders on the other hand is a prerequisite for predictive, preventive and personalised medicine in the multidisciplinary fields of dental and oral health care [48–52].

#### Environmental factors in a sensitive balance between health and disease

There is a highly sensitive interplay between a genetic component, epigenetic regulations and environmental factors that determines a sensitive balance between health and disease in individuals. Unfortunately, environment is still a largely neglected topic in health care. PPPM approach aims to develop an appropriate knowledge and technological skills for promoting affordable strategies in the emerging fields of environmental risk factors, epidemiology, healthy lifestyle, individualised nutrition, food technology and culture in a framework of cost-effective health care [29, 47, 53–55].

#### Robust PPPM platform to advance regenerative medicine

Prediction and personalisation in regenerative medicine are prerequisites for improved individual outcomes. Hence, in order to optimally match the donor to recipient and assess individual risks, a successful transplantation requires valid pathology-specific pre- and post-transplantation biomarker panels tailored to the individual. Long waiting lists of patients worldwide reflect major problems and current deficits, which require PPPM-related solutions advancing this medical area on the global scale [6]. Individual components of the overall management leading to substantially increased allograft survival and decreased patient morbidity are an improved donor-recipient matching, individual risk assessment for chronic allograft damage, prediction of graft accommodation and creation of personalised immunosuppressive algorithms.

#### Body culture and sports medicine (BCSP) effectively promoted by PPPM

PPPM strategies in BCSP are based on optimisation of the relationship between individual genetic predispositions and modifiable risk factors (nutrients, physical activity, lifestyle, etc.). Therefore, the main tools are individualised physical exercises and therapy algorithms, healthy balance between body tension and relaxation, optimised sleep algorithms according to individual circadian rhythm, innovative rehabilitation approaches, amongst others. Anti-doping control and effective measures are mandatory for PPPM implementation in advanced BCSP. High-quality research based on measurable effects utilising multilevel biomarker panels is effectively promoted by PPPM in BCSP with a particular focus on individually tailored interventions [56–59].

#### Translational medicine: a powerful bridge between PPPM science and implementation

There are many scientific fields which, on a daily basis, provide a great knowledge potentially useful for advanced medical services. However, a number of scientific articles and valuable patents remain unused. The “bottleneck” between the sciences and application has many reasons including economic circumstances and missing political regulations. In order to effectively promote the translational medicine as the “catalyser” for practical implementation of the accumulated scientific achievements, EPMA creates a robust platform for an effective dialogue between PPPM relevant professional groups on the one side, and industry and policy-makers on the other side—for more information, see the main documents of the association [6, 35]. The main goal is to translate knowledge from studies at the bench side to care at the bedside by following mechanism: from discovery to health application, to evidence-based guidelines, to advanced health care services and finally to health impacts for the patient [60, 61].

#### Information and communication technologies (ICT) resulting in cost-effective modernisation of health care

A holistic presentation of individuals and discoursed health condition by ICT approach implies a redesign of health care services. The ICT support is the prerequisite for an effective PPPM by disease modelling, individualised patient profiles, optimised diagnostic and treatment approaches. The ICT tools include mathematical modelling methods, such as probabilistic relational models and process models, prediction of a disease development, precise patient stratification, creation of the multimodal diagnostic approaches, elaboration of the best possible therapy algorithms, an estimation of individual outcomes, distanced patient monitoring, advanced avatar technologies, and bid data management amongst others. Contextually, ICT is anticipated to result in profound and cost-effective modernisation of health care benefiting the patient, health care providers and society at large [28, 29, 62–66].

#### The crucial role of multilevel diagnostics in PPPM

Accumulating evidence demonstrates that an ideal biomarker does not exist. The role of multilevel diagnostics is to provide maximum clinically relevant information by utilising pathology- and stage-specific biomarker panels at the level of medical imaging, subcellular imaging, multi-omics and relevant hybrid technologies. Integrating this information allows for targeted prevention and personalised treatment regimes, avoiding unnecessary drug toxicity, decreasing negative side-effects and reducing morbidity [27–29, 62, 67–69].

#### Laboratory medicine in PPPM concepts: from passive assistance to active advising

Delayed intervention, untargeted medication, overdosed patients and ineffective treatments, amongst others, are the deficits in currently pursued medical services that demand a revised role of laboratory medicine in health care systems. The laboratory services should become more complex, advancing multifactorial analysis. Such a complex analytics should result in recommendations and active advising for clinicians in order to more accurately interpret health-related data of the individual/patient. Therefore, an effective ICT support (see the “Information and communication technologies (ICT) resulting in cost-effective modernisation of health care” section) is mandatory. Practical implementation of novel and complex laboratory tests certainly should be considered from the viewpoint of their reasonability, cost-effectiveness and value added to a data interpretation. Smart laboratory investigation strategies and all-encompassing data interpretation are essential for an appropriate relationship between laboratory medicine and clinicians acting hand-in-hand as the decision makers responsible for better individual outcomes [70–75].

#### Well-regulated biobanking and biopreservation is pivotal for future progress in PPPM

For the future progress in development of novel biomarker panels, predictive and prognostic technologies and personalisation of treatment regimes, an internationally valid biobanking and biopreservation are essential. A proper creation of that is currently an ongoing process in PPPM [66]. Considering individual types of biological material (tissue, saliva, blood and cell samples, DNA, RNA, proteins, metabolites, etc.), the major challenges are due to:

- Consideration of ethical aspects including privacy- and security-related issues [76, 77]
- Adequate national and international regulations
- Optimised protocols for collecting, storing and retrieving the samples
- High analytical quality of all the process of biobanking and biopreservation
- Adequately organised clinical/patient databases.

An effective support by the advanced ICT systems for smoothly run processing and adequate data interpretation is crucial for the clinical utility of biobanks [66].

#### Design of professional interactome in PPPM

PPPM carries highly multi- and interdisciplinary character and demonstrates high level of international cooperation. Consequently, related networking demands an effective interaction amongst professional groups as well as between health care professionals and patient groups and policy-makers. All these groups currently do “speak different languages,” which may create some communication barriers, however, reinforcing each group’s perspective to reach higher level of understanding and cooperation in PPPM framework. The specific output of this design activity is the so-called professional interactome [78]. The PPPM-related interactome represents the most optimal model of health care organisation with significantly increased quality of multilevel communication and cooperation resulting in improved individual patient outcomes and health care economy (see the “Advanced business models for PPPM concepts in health care” section).

#### Education as the heart of the PPPM-related scientific excellence and successful practical implementation

The ultimate goal is to create a new culture in the health care sector and to promote high level of professionalism by new generations of healthcare-givers who will be capable to implement an all-encompassing approach to patient care recognising the complexity and individuality of the human being. In order to promote innovative educational programmes, the following worldwide pioneer initiatives have been developed:

- The EPMA Journal regularly updates information about medical innovations and advanced health care providing expert recommendations in predictive diagnostics, targeted preventive measures and individualised treatment algorithms (https://epmajournal.biomedcentral.com/ and http://www.springer.com/biomed/journal/13167).
- Advances in predictive, preventive and personalised medicine (http://www.springer.com/series/10051): this book series, launched in 2012, provides an overview of complex strategies, innovative technologies, novel biomarker panels, and multidisciplinary aspects of advanced biomedical approaches in individual PPPM areas and health care as a whole. New technologies and guidelines are provided for medical ethics, early and predictive diagnostics, targeted prevention, treatments tailored to the person, health care organisation and economy. This book series is intended to serve as a reference source for multidisciplinary research and the health care industry with special emphasis on advanced health promotion and cost-effective treatment of diseases.

#### Advanced business models for PPPM concepts in health care

If left unchanged, a long-term poor cost-effectiveness may lead to economic collapse of current health care systems with persisting archaic business models. Across Europe, there is a great diversity of systems, payment models and reimbursement schemes in health care [72]. This imposes a highly fragmented market. On the one hand, there is a need for policy dialogue in order to achieve improved structure and delivery. On the other hand, advanced business models are required, in order to motivate

- Healthcare-givers to apply more individualised diagnostic and treatment approaches
- Healthy individuals and patients to accept greater responsibility towards their own health condition
- Industry to create novel products for health support, promotion and monitoring
- Policy-makers for smart long-term regulations in health care sector such as an effective promotion of increased health literacy in population, advanced screening programmes and new reimbursement models for individual subpopulations and professional groups
- Finally, the society at large to reinvest budgets focused on the most cost-effective health promotion and primary health care.

In view of economic strain and the ageing populations, PPPM-related innovation in health care systems is critical for keeping the high quality of health care affordable and sustainable on European and global scale. Since its very beginning, EPMA is systematically working on the economy of PPPM that is pivotal for advancing health care on European and global scale [6, 8, 20–22, 28, 30, 35, 37, 40, 71, 72, 79–82].

## Conclusions and expert recommendations

Concluding remarks are summarised in Table 1 in form of advantages and limitations listed for individual types of medicines analysed in this paper followed by recommendations for their most optimal application.

**Table 1:** Conclusions and expert recommendations

Term	Advantages	Limitations	Optimal application and unique niche
Traditional, complementary and alternative medicine, TCAM	Increases the own repair capacity of the human body; deals with natural products and physiological approaches; is dedicated to disease prevention and well-being; highly effective at the level of suboptimal health condition	Less effective for disease care; in manifest pathologies can be applied to complement conventional treatments such as surgery, chemo-therapy, etc.; cultural barriers can exist when TCAM is introduced by the country of origin to other countries with sufficiently different cultural habits	➢ Diagnosis and treatment of suboptimal health conditions ➢ Cost-effective preventive medicine ➢ Emphasises well-being ➢ Pain management ➢ Complementary treatments ➢ Cultural traditions of the country of origin
Person-centred medicine, PCM	Promotion of health as a state of physical, mental, social and spiritual well-being; potential for disease reduction; emphasis on science and humanism; PCM promotes approaches to health improvement, respect and responsibility at individual and community levels	Realisation of the ideals promoted by PCM demands clear definitions and validated strategies	➢ Health care philosophy ➢ Mental maturation of society at large ➢ Integration of sciences and humanism ➢ Promotion of health ➢ Promotion of respect and responsibility in the society
Individualised medicine, IM	IM propagates a holistic approach by acknowledging multidimensional interaction between internal and external risk factors which vary from individual to individual.	IM is clearly focused on individualisation of standardised intervention, but it provides less developed concepts of predictive and preventive medicine, if any.	➢ Holistic approach to standardised intervention ➢ Patient categorisation and modelling
Stratified medicine, SM	More targeted treatments according to individual patient subtype; stratified treatment algorithms	Although being an extremely important instrument, SM represents just one step towards “personalised medicine.”	➢ Cohort subgrouping ➢ Patient stratification
Personalised medicine, PM	Actually considered best possible medical treatments adapted to the needs of the patient	Concepts of PM are adapted to “disease care” but not to “health care.”	Semi-personalised medicine compromising between standardisation and individualisation in medicine
Precision medicine, PrecMed	PrecMed attracts attention of policy-makers to problems persisting in medical services; additionally released budgets in medical sciences; increased publicity for disease care; potentially increased cooperation level between individual medical fields	Politically motivated initiative utilising advantages of already existing and above listed approaches; strong limitations by selectively promoted technological focuses (e.g. genomics); unclear integration strategies in medicine; unclear cost-effectiveness, benefits to individual patient cohorts and overall health care economy	➢ Potentially improved clinical impacts of specific areas such as genomics ➢ Potential technological integration in medical fields ➢ Potentially improved outcomes in some patient cohorts
Predictive preventive and personalised medicine, PPPM	PPPM is a really complex all-encompassing approach combining advantages of the above listed individual approaches and minimising their specific disadvantages; clear concepts demonstrating the highest level of maturity; the most optimal strategies considering interests of healthy individuals, subpopulations, patient cohorts, health care systems and society as a whole.	PPPPM is considered as the “medicine of the future” which needs the paradigm change for entire spectrum of medical research and services, improved professional and general educational levels, new economic and application models for both disease and health care.	➢ Desirable versus current health care systems ➢ Predictive medicine ➢ New spectrum of screening programmes ➢ Targeted prevention ➢ Currently unmet needs of healthy subpopulations and patient cohorts ➢ Cost-effective medical services and optimised health care economy ➢ New dimension of professional interests ➢ New scale of the knowledge integration ➢ Highly motivated technological innovation ➢ Highly motivated interdisciplinary and multidisciplinary cooperation ➢ Individualised patient profiling ➢ Active participation of patients in the health care process

## Abbreviations

CVD: Cardiovascular disease
DM: Diabetes mellitus
EPMA: European Association for Predictive, Preventive and Personalised Medicine
IM: Individualised medicine
NNND: Neurological, neuropsychiatric and neurodegenerative diseases
PCM: Person-centred medicine
PM: Personalised medicine
PPPM: Predictive, preventive and personalised medicine
PrecMed: Precision medicine
RD: Rare disease
SM: Stratified medicine
TCAM: Traditional, complementary and alternative medicine
